# Supplementary material for: Validation in an Independent Cohort of MiR-122, MiR-1271, and MiR-15b as Urinary Biomarkers for the Potential Early Diagnosis of Clear Cell Renal Cell Carcinoma
Source: Cancers (Basel). 2022 Feb 22;14(5):1112. doi: 10.3390/cancers14051112 (PMC8909007; doi:10.3390/cancers14051112)
Supplement: Supplementary file 1 [file cancers-14-01112-s001.zip › cancers-1536048 supplementary.pdf]

cancers

MDPI

Supplementary Material

Validation in an Independent Cohort of MiR-122, MiR-1271, and MiR-15b as Urinary Biomarkers for the Potential Early Diagnosis of Clear Cell Renal Cell Carcinoma

Giovanni Cochetti [1], Luigi Cari [2], Vincenza Maulà [1], Rosy Cagnani [1], Alessio Paladini [1], Michele Del Zingaro [1], Giuseppe Nocentini [2,*] and Ettore Mearini [1]

[1] Division of Urology Clinic, Department of Medicine and Surgery, University of Perugia, 06129 Perugia, Italy; giovanni.cochetti@unipg.it (G.C.); vincenza.maula@unipg.it (V.M.); rosy.cagnani@unipg.it (R.C.); alessio.paladini@ospedale.perugia.it (A.P.); michele.delzingaro@unipg.it (M.D.Z.); ettore.mearini@unipg.it (E.M.)
[2] Pharmacology Section, Department of Medicine and Surgery, University of Perugia, 06129 Perugia, Italy; luigi.cari@unipg.it (L.C.)
* Correspondence: giuseppe.nocentini@unipg.it; Tel.: +39-07-5585-8126

Table S1. Ct values of miR-122-5p, miR-1271-5p, miR-15b-5p, Cel-miR-39-3p, miRTC, and miR-16-5p, obtained using the urine of HSs and patients with ccRCC.

	Ct Value	miR-122-5p	miR-1271-5p	miR-15b-5p	Cel-miR-39-3p	miRTC	miR-16-5p
ccRCC	**ccRCC 1**	40.00	38.10	32.85	28.41	32.19	39.21
	**ccRCC 2**	29.24	29.42	28.05	19.97	19.11	27.20
	**ccRCC 3**	34.76	30.02	35.24	22.85	28.94	30.69
	**ccRCC 4**	39.47	33.29	31.19	23.56	28.16	31.76
	**ccRCC 5**	35.30	29.53	37.15	30.10	29.32	27.04
	**ccRCC 6**	30.06	29.32	28.73	22.95	22.83	25.09
	**ccRCC 7**	36.62	31.08	40.00	23.80	23.95	31.21
	**ccRCC 8**	35.54	33.21	35.18	27.49	24.93	30.79
	**ccRCC 9**	33.75	30.70	33.38	29.05	26.81	30.14
	**ccRCC 10**	37.37	29.98	40.00	32.54	32.57	30.32
	**ccRCC 11**	38.63	31.31	38.45	24.82	32.45	37.21
	**ccRCC 12**	35.85	29.98	39.54	26.52	23.36	32.10
	**ccRCC 13**	40.00	32.33	39.32	23.67	26.55	30.10
	**ccRCC 14**	39.20	31.94	40.00	26.60	25.95	35.12
	**ccRCC 15**	38.95	30.35	38.63	26.76	30.19	30.60
	**ccRCC 16**	38.26	34.57	38.86	29.72	33.23	30.89
	**ccRCC 17**	31.14	29.58	28.03	34.16	31.09	29.30
	**ccRCC 18**	38.71	30.76	35.01	26.63	28.89	28.40
	**ccRCC 19**	25.50	24.51	28.01	23.24	24.02	23.43
	**ccRCC 20**	24.69	24.93	26.95	19.62	19.32	23.00
	**ccRCC 21**	31.20	28.12	25.59	21.51	21.04	25.45
	**ccRCC 22**	36.00	30.20	25.88	18.40	21.27	26.33
	**ccRCC 23**	35.31	29.28	26.12	20.90	23.80	30.24
	**ccRCC 24**	30.99	29.53	29.73	33.62	32.30	29.24
**Mean ccRCC**		**34.86**	**30.50**	**33.41**	**25.70**	**26.76**	**29.79**
**SD ccRCC**		**4.43**	**2.77**	**5.31**	**4.38**	**4.42**	**3.87**
HSs	**HS 1**	36.65	30.18	38.24	29.94	39.18	26.48
	**HS 2**	31.29	29.69	25.44	27.69	31.17	25.23
	**HS 3**	40.00	33.38	40.00	31.82	34.89	32.63
	**HS 4**	39.00	31.03	39.02	27.02	33.44	36.15
	**HS 5**	39.05	33.16	37.96	31.51	34.67	32.59

	**HS 6**	40.00	36.37	40.00	23.18	28.40	38.06
	**HS 7**	38.39	30.58	34.39	29.52	36.60	30.22
	**HS 8**	34.93	32.23	29.67	30.32	37.72	28.57
	**HS 9**	36.28	31.54	38.56	28.91	34.20	31.07
	**HS 10**	35.92	31.15	38.84	25.09	26.54	29.08
	**HS 11**	40.00	31.37	39.00	31.96	27.65	31.12
	**HS 12**	40.00	28.87	38.36	28.26	24.23	35.32
	**HS 13**	35.87	29.14	27.26	17.70	23.15	26.59
	**HS 14**	31.19	32.67	30.98	30.60	39.72	33.10
	**HS 15**	35.60	29.25	40.00	34.52	36.85	30.59
	**HS 16**	37.86	30.40	35.31	31.30	37.11	31.92
	**HS 17**	40.00	30.30	39.21	34.95	34.45	31.34
	**HS 18**	34.55	33.38	38.32	20.77	32.07	28.54
	**HS 19**	40.00	32.39	40.00	24.49	32.61	31.44
	**HS 20**	40.00	31.66	26.74	32.35	31.28	28.17
	**HS 21**	40.00	30.41	38.05	33.70	40.00	32.11
	**HS 22**	33.80	34.35	40.00	24.37	27.65	39.51
	**HS 23**	40.00	35.25	40.00	26.64	28.50	36.07
**Mean HSs**		**37.41**	**31.68**	**36.32**	**28.55**	**32.70**	**31.56**
**SD HSs**		**2.86**	**1.97**	**4.78**	**4.40**	**4.90**	**3.66**

Table S2. Ct and ΔCt of the seven parameters #1, #2, #3, #4, #5, #6, and #7. ΔCt (parameters #2, #3, #4, #5, #6, and #7) were calculated using data reported in Table S1.

	**Parameter**	**# 1**	**# 2**	**# 3**	**# 4**	**# 5**	**# 6**	**# 7**
	**Parameter Description**	**miR-1271-5p**	**miR-122-5p/miR-16-5p**	**miR-122-5p/miRTC**	**miR-1271-5p/miR-16-5p**	**miR-1271-5p/miRTC**	**miR-15b-5p/miRTC**	**miR-15b-5p/Cel-miR-39-3p**
		**Ct**	**ΔCt**	**ΔCt**	**ΔCt**	**ΔCt**	**ΔCt**	**ΔCt**
**ccRCC**	**ccRCC 1**	38.10	0.79	7.81	- 1.11	5.91	0.66	4.44
	**ccRCC 2**	29.42	2.04	10.13	2.22	10.31	8.94	8.08
	**ccRCC 3**	30.02	4.07	5.82	−0.67	1.08	6.30	12.39
	**ccRCC 4**	33.29	7.71	11.31	1.53	5.13	3.03	7.63
	**ccRCC 5**	29.53	8.27	5.98	2.49	0.21	7.83	7.05
	**ccRCC 6**	29.32	4.97	7.23	4.23	6.49	5.90	5.78
	**ccRCC 7**	31.08	5.41	12.67	−0.13	7.13	16.05	16.20
	**ccRCC 8**	33.21	4.75	10.61	2.42	8.28	10.25	7.69
	**ccRCC 9**	30.70	3.61	6.94	0.56	3.89	6.57	4.33
	**ccRCC 10**	29.98	7.05	4.80	−0.34	−2.59	7.43	7.46
	**ccRCC 11**	31.31	1.42	6.18	−5.90	−1.14	6.00	13.63
	**ccRCC 12**	29.98	3.75	12.49	−2.12	6.62	16.18	13.02
	**ccRCC 13**	32.33	9.90	13.45	2.23	5.78	12.77	15.65
	**ccRCC 14**	31.94	4.08	13.25	−3.18	5.99	14.05	13.40
	**ccRCC 15**	30.35	8.35	8.76	−0.25	0.16	8.44	11.87
	**ccRCC 16**	34.57	7.37	5.03	3.68	1.34	5.63	9.14
	**ccRCC 17**	29.58	1.84	0.05	0.28	−1.51	−3.06	−6.13
	**ccRCC 18**	30.76	10.31	9.82	2.36	1.87	6.12	8.38
	**ccRCC 19**	24.51	2.07	1.48	1.08	0.49	3.99	4.77
	**ccRCC 20**	24.93	1.69	5.37	1.93	5.61	7.63	7.33
	**ccRCC 21**	28.12	5.75	10.16	2.67	7.08	4.55	4.08
	**ccRCC 22**	30.20	9.67	14.73	3.87	8.93	4.61	7.48
	**ccRCC 23**	29.28	5.07	11.51	−0.96	5.48	2.32	5.22
	**ccRCC 24**	29.53	1.75	-1.31	0.29	−2.77	−2.57	−3.89
**Mean ccRCC**		**30.50**	**5.07**	**8.09**	**0.72**	**3.74**	**6.65**	**7.71**
**SD ccRCC**		**2.77**	**2.94**	**4.27**	**2.37**	**3.83**	**4.94**	**5.33**
**HSs**	**HS 1**	30.18	10.17	−2.53	3.70	−9.00	−0.93	8.30
	**HS 2**	29.69	6.06	0.12	4.46	−1.48	−5.73	−2.25
	**HS 3**	33.38	7.37	5.11	0.75	−1.51	5.11	8.18
	**HS 4**	31.03	2.85	5.56	−5.12	−2.41	5.58	12.00

	**HS 5**	33.16	6.46	4.38	0.57	−1.51	3.29	6.45
	**HS 6**	36.37	1.94	11.60	−1.69	7.97	11.60	16.82
	**HS 7**	30.58	8.17	1.79	0.36	−6.02	−2.21	4.87
	**HS 8**	32.23	6.36	−2.79	3.66	−5.49	−8.05	−0.65
	**HS 9**	31.54	5.21	2.08	0.47	−2.66	4.36	9.65
	**HS 10**	31.15	6.84	9.38	2.07	4.61	12.30	13.75
	**HS 11**	31.37	8.88	12.35	0.25	3.72	11.35	7.04
	**HS 12**	28.87	4.68	15.77	−6.45	4.64	14.13	10.10
	**HS 13**	29.14	9.28	12.72	2.55	5.99	4.11	9.56
	**HS 14**	32.67	−1.91	−8.53	−0.43	−7.05	−8.74	0.38
	**HS 15**	29.25	5.01	−1.25	−1.34	−7.60	3.15	5.48
	**HS 16**	30.40	5.94	0.75	−1.52	−6.71	−1.80	4.01
	**HS 17**	30.30	8.66	5.55	−1.04	−4.15	4.76	4.26
	**HS 18**	33.38	6.01	2.48	4.84	1.31	6.25	17.55
	**HS 19**	32.39	8.56	7.39	0.95	−0.22	7.39	15.51
	**HS 20**	31.66	11.83	8.72	3.50	0.38	−4.54	−5.61
	**HS 21**	30.41	7.89	0	−1.70	−9.59	−1.95	4.35
	**HS 22**	34.35	−5.71	6.15	−5.16	6.70	12.35	15.63
	**HS 23**	35.25	3.93	11.50	−0.82	6.75	11.50	13.36
**Mean HSs**		**31.68**	**5.85**	**4.71**	**0.12**	**−1.01**	**3.62**	**7.77**
**SD HSs**		**1.97**	**3.86**	**5.98**	**3.03**	**5.45**	**6.86**	**6.24**
**Mean ccRCC/Mean HSs**		1.18	0.78	−3.39	−0.59	−4.75	−3.03	0.06
**Passed KS test**		No	Yes	Yes	Yes	No	Yes	Yes
***p* value**		0.0645	0.4408	0.0300	0.4578	0.0025	0.0881	0.9704
**Range of values shown by ccRCC patients**	**Range–SD**	27.73	2.13	3.82	−1.65	−0.09	1.71	2.38
	**Range + SD**	33.27	8.01	12.37	3.08	7.57	11.59	13.04

Table S3. Derivative of Ct and ΔCt values of the seven parameters obtained as described in the Materials and Methods section and their sum. In bold the values which are below the disease cut-off (−14.76).

	**Parameter**	**# 1 [1]**	**# 2 [1]**	**# 3 [2]**	**# 4 [2]**	**# 5 [2]**	**# 6 [2]**	**# 7 [1]**	**Sum of parameters**	**Diseased cut-off value**
	**ccRCC 3**	−3.25	−3.94	−2.00	−0.98	−1.17	−4.59	−0.65	**−16.57**	
	**ccRCC 4**	0.02	−0.30	−7.49	−3.18	−5.22	−1.32	−5.41	**22.89**	
	**ccRCC 5**	−3.74	0.26	−2.16	−4.41	−0.30	−6.12	−5.98	**−22.19**	
	**ccRCC 6**	−3.95	−3.04	−3.41	−5.88	−6.58	−4.19	−7.26	**−34.30**	
	**ccRCC 7**	−2.19	−2.60	−8.85	−1.52	−7.22	−14.34	3.16	**−33.55**	
	**ccRCC 8**	−0.06	−3.26	−6.79	−4.07	−8.37	−8.54	−5.35	**−36.43**	
**S**	**ccRCC 9**	−2.57	−4.40	−3.12	−2.21	−3.98	−4.86	−8.71	**−29.84**	
**A**	**ccRCC 10**	−3.29	−0.96	−0.98	−1.31	2.50	−5.72	−5.58	**−15.33**	
**M**	**ccRCC 11**	−1.96	−6.59	−2.36	4.25	1.05	−4.29	0.59	−9.30	
**P**	**ccRCC 12**	−3.29	−4.26	−8.67	0.47	−6.71	−14.47	−0.02	**−36.94**	
**L**	**ccRCC 13**	−0.94	1.89	−9.63	−3.88	−5.87	−11.06	2.61	**−26.87**	
**E**	**ccRCC 14**	−1.33	−3.92	−9.43	1.53	−6.08	−12.34	0.36	**−31.21**	**−14.76**
	**ccRCC 15**	−2.92	0.34	−4.94	−1.40	−0.25	−6.73	−1.17	**−17.06**	
**C**	**ccRCC 16**	1.30	−0.64	−1.21	−5.33	−1.43	−3.92	−3.90	**−15.12**	
**O**	**ccRCC 17**	−3.69	−6.17	3.77	−1.93	1.42	4.77	−19.17	**−20.99**	
**D**	**ccRCC 18**	−2.51	2.30	−6.00	−4.01	−1.96	−4.41	−4.66	**−21.24**	
**E**	**ccRCC 19**	−8.76	−5.94	2.34	−2.73	−0.58	−2.28	−8.27	**−26.21**	
	**ccRCC 20**	−8.34	−6.32	−1.55	−3.58	−5.70	−5.92	−5.71	**−37.11**	
	**ccRCC 21**	−5.15	−2.26	−6.34	−4.32	−7.17	−2.84	−8.96	**−37.03**	
	**ccRCC 22**	−3.07	1.66	−10.91	−5.52	−9.02	−2.90	−5.56	**−35.31**	
	**ccRCC 23**	−3.99	−2.94	−7.69	−0.69	−5.57	−0.61	−7.82	**−29.30**	
	**ccRCC 24**	−3.74	−6.26	5.13	−1.94	2.68	4.28	−16.93	**−16.77**	
	**HS 1**	−3.09	2.16	6.35	−5.35	8.90	2.64	−4.73	6.89	

**HS 2**	−3.58	−1.95	3.70	−6.11	1.39	7.44	−15.29	−14.39
**HS 3**	0.11	−0.64	−1.29	−2.40	1.42	−3.40	−4.86	−11.05
**HS 4**	−2.24	−5.16	−1.74	3.47	2.32	−3.87	−1.04	−8.25
**HS 5**	−0.11	−1.55	−0.56	−2.22	1.42	−1.58	−6.59	−11.18
**HS 6**	3.10	−6.07	−7.78	0.04	−8.06	−9.89	3.78	**−24.87**
**HS 7**	−2.69	0.16	2.04	−2.01	5.93	3.92	−8.17	−0.82
**HS 8**	−1.04	−1.65	6.61	−5.31	5.40	9.76	−13.69	0.09
**HS 9**	−1.73	−2.80	1.74	−2.12	2.57	−2.65	−3.39	−8.37
**HS 10**	−2.12	−1.17	−5.56	−3.72	−4.70	−10.59	0.71	**−27.14**
**HS 11**	−1.90	0.87	−8.53	−1.90	−3.81	−9.64	−6.00	**−30.90**
**HS 12**	−4.40	−3.32	−11.95	4.80	−4.73	−12.42	−2.94	**−34.96**
**HS 13**	−4.13	1.27	−8.90	−4.20	−6.08	−2.40	−3.48	**−27.91**
**HS 14**	−0.60	−9.92	12.35	−1.22	6.96	10.45	−12.66	5.37
**HS 15**	−4.02	−3.00	5.07	−0.31	7.51	−1.44	−7.56	−3.74
**HS 16**	−2.87	−2.07	3.07	−0.13	6.62	3.51	−9.03	−0.89
**HS 17**	−2.97	0.65	−1.73	−0.61	4.06	−3.05	−8.78	−12.42
**HS 18**	0.11	−2.00	1.34	−6.49	−1.40	−4.54	4.51	−8.46
**HS 19**	−0.88	0.55	−3.57	−2.60	0.13	−5.68	2.47	−9.57
**HS 20**	−1.61	3.82	−4.90	−5.15	−0.47	6.25	−18.65	**−20.70**
**HS 21**	−2.86	−0.12	3.82	0.05	9.50	3.66	−8.69	5.37
**HS 22**	1.08	−13.72	−2.33	3.51	−6.79	−10.64	2.59	**−26.29**
**HS 23**	1.98	−4.08	−7.68	−0.83	−6.84	−9.79	0.32	**−26.91**

[1] Values were calculated with the following formula: subject Ct (or ΔCt) −[mean Ct (or ΔCt) of ccRCC + SD of ccRCC].

[2] Values were calculated with the following formula: [mean Ct (or ΔCt) of ccRCC −SD of ccRCC] −subject Ct (or ΔCt).

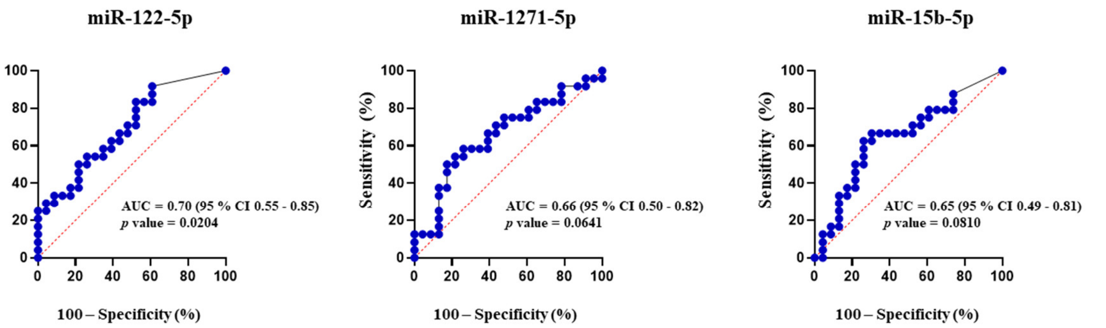

(a)

Sensitivity	83% (95 % CI, 64% - 93%)
Specificity	48% (95 % CI, 29% - 67%)

(b)

Sensitivity	83% (95 % CI, 64% - 93%)
Specificity	35% (95 % CI, 19% - 55%)

(c)

Sensitivity	75% (95 % CI, 55% - 88%)
Specificity	43% (95 % CI, 26% - 63%)

Figure S1. ROC curves of miR-122-5p (**a**), miR-1271-5p (**b**), and miR-15b-5p (**c**).

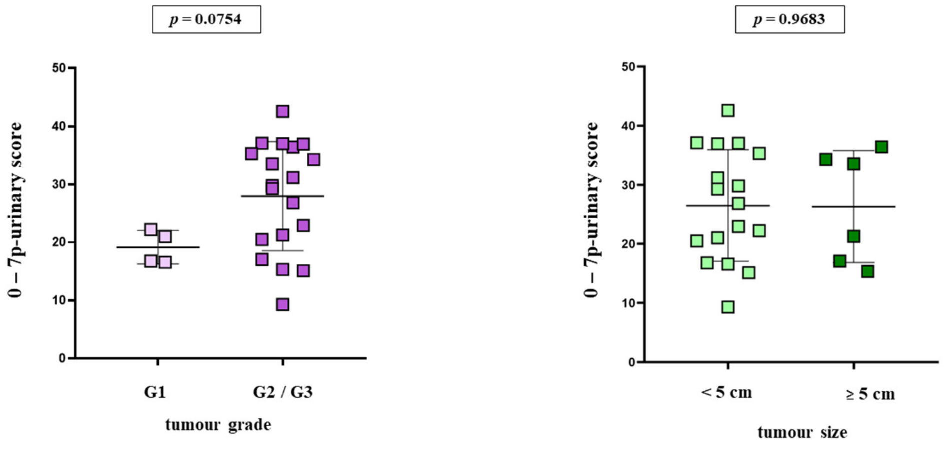

(a) (b)

Figure S2. Lack of difference of the 7p-urinary scores between ccRCC tumors of different grades and sizes. (**a**) The 7p-urinary score mean of patients with grade 1 (G1) ccRCC is not significantly different (unpaired t-test) from that of patients with ccRCC with grade 2 and grade 3 (G2 – G3). (**b**) The 7p-urinary score mean of patients with ccRCC with a size smaller than 5 cm is not significantly different (unpaired t-test) from that of patients with a ccRCC size greater than 5 cm.
